# Soil environmental changes drive root decomposition under acid and nitrogen deposition in Chinese fir plantation, China

**DOI:** 10.1186/s12870-026-08816-z

**Published:** 2026-04-25

**Authors:** Xuanran Yu, Chang Pan, Jinchi Zhang, Xingyu Zhang, Aodeng Rong, Qingguo Tong, Xiongfei Zhang, Chong Li, Hui Nie, Jingyi Zeng, Yangyang Wang, Xin Liu

**Affiliations:** 1 Co-Innovation Center for Sustainable Forestry in Southern China of Jiangsu Province, Key Laboratory of Soil and Water Conservation and Ecological Restoration of Jiangsu Province, Nanjing Forestry University, Nanjing, 210037 China; 2 State Key Laboratory of Nutrient Use and Management, College of Resources and Environmental Sciences, China Agricultural University, Beijing, 100193 China; 3 Inner Mongolia Big Data Center, Hohhot, 010090 China; 4 CNBM Mining Investment (Jiangsu) Co., Ltd, Nanjing, China; 5 Department of Renewable Resources, University of Alberta, Edmonton, AB T6G 2E3 Canada

**Keywords:** Acid rain, Nitrogen deposition, Root chemistry, Soil environment

## Abstract

The decomposition of underground roots is a crucial component of soil carbon turnover and nutrient input processes. However, our understanding of how root chemistry and soil environments interact during root decomposition remains limited, and differences in the decomposition processes among different root orders are still unclear. Therefore, this study was conducted using Chinese fir (*Cunninghamia lanceolata*) as the experimental subject. We combine root chemistry-only change, soil environment-only change, and in-situ experiments to investigate the effects of root chemistry and soil environments during root decomposition, as well as the differences in decomposition processes among different root orders under the context of acid rain and nitrogen deposition. Our findings indicate that the soil environment has a long-term and more significant influence on root decomposition. In the SHAP analysis of relative contributions, soil environmental factors explained 61.2% of the variation in low-order roots and 55.9% of the variation in high-order roots. The root decomposition process primarily shifts from being dominated by root chemistry in the short term to being governed by the soil environment in the long term. Different root orders show similar changing trends but differ in their timing of change: for low-order, the dominant factor shifts to the soil environment after 6 months, whereas for high-order, this shift occurs after 10 months. The influence of the soil environment is mediated through soil nutrient. For instance, high-order roots are primarily driven by the soil carbon-nitrogen ratio, with a direct effect of 0.38, while low-order roots are dominated by available potassium, with a direct effect of 0.57. Interestingly, under the context of acid rain and nitrogen deposition, soil environment and root chemistry showed a synergistically enhancing trend in inhibiting the decomposition of low-order roots, whereas for high-order roots, the two factors exhibited an opposite trend of mutually weakening inhibition. In summary, this study highlights the long-term importance of the soil environment in root decomposition and the varying responses of different root orders, contributing to a deeper understanding of root decomposition and soil carbon turnover processes in forest ecosystems.

**Supplementary Information:**

The online version contains supplementary material available at 10.1186/s12870-026-08816-z.

## Introduction

In forest ecosystems, litter decomposition links plant carbon allocation to the soil carbon pool, and drives global carbon and nutrient cycling [1]. Among these processes, the decomposition of underground roots accounts for approximately 48% of total litter input [2].and provides stable nutrient inputs [3]. Furthermore, the majority of carbon in the soil carbon pool originates from underground roots [4], with root turnover alone contributing 25% to 80% of the total soil carbon annually [5], along with 10% to 75% of nitrogen inputs [6]. Root decomposition regulates the formation of soil organic matter [7, 8], nutrient supply and ecosystem stability, especially in degraded forests. Chinese fir is a major timber species in southern China, but plantation degradation threatens regional ecosystem health. Root decomposition is critical for soil fertility and function recovery in degraded Chinese fir forests [9], yet its regulatory mechanisms remain unclear.

Existing studies indicate that root decomposition is jointly influenced by root chemistry and soil environment [10, 11]. Root chemistry is a core intrinsic factor influencing its decomposition process [10, 12, 13]. Nitrogen content [14] and phosphorus content [15] are generally positively correlated with the decomposition rate [15, 16], whereas lignin content shows a negative correlation [11]. In relevant studies on cover crops in farmland, the higher root C: N ratio and biomass of yellow mustard roots accelerated their decomposition rate. In particular, the initial lignification degree of roots is considered the best predictor of decomposition rates [17]. Soil environment is also a critical external factor regulating root decomposition [11]. Soil pH, moisture, and acid rain type, among other factors, can significantly influence the decomposition process [18, 19]. They can also indirectly regulate root decomposition by altering microbial community structure and function (such as fungal composition and abundance) [20, 21]. Notably, root functional traits exhibit significant hierarchical differences. Traditional studies often categorize roots with diameters less than 2 mm as “fine roots,” but in reality, roots consist of multiple branching orders, each with distinct morphological, chemical, and functional characteristics [22, 23]. This heterogeneity means that traditional diameter-based classifications may fail to accurately reflect functional differences, potentially leading to biases in root turnover estimates [24] and hindering the understanding of the true regulatory mechanisms of root decomposition.

In recent years, Chinese fir forests in southern China have faced the dual pressures of acid rain and nitrogen deposition [25]. These environmental changes directly cause soil acidification [19, 26, 28], alter soil carbon sequestration capacity and microbial community structure [20, 21] and affect root chemistry. However, most existing large‑scale studies on the effects of nitrogen deposition on plant roots lack analyses of root decomposition differences among distinct root orders [29, 30]. Against this context, key scientific questions arise: In the root decomposition process of degraded Chinese fir forests, which factor plays a more critical role—root chemistry or the soil environment? Furthermore, does this regulatory role vary across root orders? This study accounts for the combined effects of acid rain and nitrogen deposition and investigates decomposition differences among fine‑root orders. It complements global‑scale root decomposition research [31] by exploring decomposition mechanisms of different root orders in degraded plantation ecosystems. Experimental plots were established in a Chinese fir plantation at the Yangtze River Delta Forest Ecosystem Research Station, including control (CK), nitrogen deposition (N), acid rain (AS), and combined acid rain and nitrogen deposition treatments (ASN). Through three parallel experiments (root chemistry change experiments, soil environment change experiments, and in-situ experiments), a two-year study was conducted, encompassing root sample preparation and decomposition phases, with a focus on roots of different orders. The aims of this study are: (1) to clarify the relative contributions of changes in root chemistry and soil environment to root decomposition in Chinese fir under acid rain and nitrogen deposition. (2) to reveal differences in the responses of root decomposition across different root orders to these factors, thereby providing a theoretical basis for the ecological restoration of degraded Chinese fir forests.

The research hypotheses of this study include: (1) Changes in the soil environment primarily affect root decomposition by altering root chemistry. (2) There are differences in the decomposition processes between low-order and high-order roots.

## Materials and methods

### Site description

The study site is located at the Yangtze River Delta Forest Ecosystem Positioning Research Station, which is affiliated with Nanjing Forestry University at latitude 32°07′N and longitude 119°12′E in Nanjing, Jiangsu Province, China. This area belongs to a northern subtropical monsoon, with an average temperature of 15.1 °C, mean annual relative humidity of 79% and an average annual rainfall of 1,055.6 mm, mainly concentrated in the summer seasons, frost-free period of 233 days.

It is situated in Jianghuai region. The soil is classified as yellow-brown soil, with a horizon depth of 40–60 cm and the pH of the soil was range from 3.0 to 4.0. The average height of the Chinese fir trees at the site was 10.8 m; the average diameter at breast height was 20.6 cm; the density of the forest stand was 850 plants-hm^− 2^ and the tree is 40 years old.

### Sample

*Cunninghamia lanceolata* (Taxodiaceae), commonly known as Chinese fir, is an endemic and native tree species to the subtropical regions of China and also one of the most important fast-growing timber tree species. It is an evergreen coniferous tree belonging to the genus *Cunninghamia* in the family Cupressaceae. The roots of the Chinese fir samples collected in this study were taken from Chinese fir plantation managed by the Yangtze River Delta Forest Ecosystem Positioning Research Station, which is affiliated with Nanjing Forestry University. As a research team from Nanjing Forestry University, we have obtained official approval for plot establishment and sample collection, and all sampling procedures are fully conducted in compliance with local laws and regulations. Voucher specimens are stored temporarily in the Dendrological Herbarium, Nanjing Forestry University. Dr. Liu Xin, undertook the formal identification of Chinese fir roots in this experiment.

### Experimental design and sample collection

#### Sample plot setting

According to the research methods previously determined by us [32, 33], This study was conducted at the Yangtze River Delta Forest Ecosystem Positioning Research Station (32°07′N,119°12′E). Separate experimental plots for acid rain and nitrogen deposition were established within a Chinese fir plantation in 2021.The experiment of a complete random design with 4 treatments in 12 discrete plots. Each plot measured 10 m × 10 m in area. To prevent cross-contamination between treatments, PVC was inserted 40 cm into ground, extending 5.0 cm above the surface. A throughfall collector with an area of 8.33 m² was installed 5 m away from each plot (1/12 of 10 m × 10 m plot). This collector captured Chinese fir forest penetrating rainwater for acid rain simulation solutions.

Simulated acid rain was prepared using 0.5 mol·L⁻¹ H₂SO₄ and 0.5 mol·L⁻¹ HNO₃ stock solutions formulated at SO₄²⁻:NO₃⁻ molar ratios of 5:1 [9]. Following each rainfall event, the collected rainwater was used to create acid rain simulation solutions with a pH of 2.5. In our simulated nitrogen deposition experiment, a high deposition rate was employed to examine the response of the root decomposition process to this treatment. Urea was used in the simulated nitrogen deposition experiment at an addition rate of 15 g·m⁻²·yr⁻¹. For each 10 m × 10 m plot, the annual urea application totals 3217.5 g, with a monthly addition of 268.13 g. This monthly quantity is divided into two spray events, during which urea is dissolved in simulated acid rain solution for co-application. The total amount of simulated acid rain applied monthly is approximately 1/12 of the monthly average precipitation in the study area.

In general, there are four simulated treatments: control group(CKs, Chinese fir forest rain penetration), acid rain(AS, pH = 2.5),nitrogen addition(N,15 g·m^−^²·yr^− 1^),acid rain + nitrogen addition(ASN, pH = 2.5,15 g·m^−^²·yr^− 1^).The acid rain simulation solution was sprayed on the ground of the plot while collected rainwater was sprayed on the control group plot directly.

#### Sample collection

**Fig. 1:**
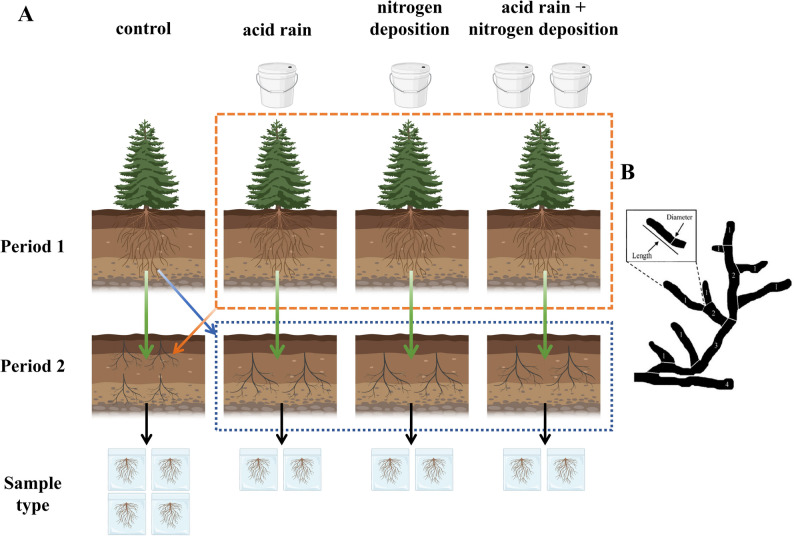
Schematic diagram of experiment. **A**: root collection and root decomposition stages. **B**: different root order classifications

This sample collection was conducted in two stages:

Sample collection stage of the root decomposition experiment (From October 2021 to October 2022): Excavate soil blocks of 20 cm × 20 cm × 10 cm at the base of the tree trunk. Dig 3 soil blocks in each plot. Carefully remove the crushed soil around the roots from the soil blocks containing root samples until the branching structure of the roots can be identified. Root grading according to Pregitzer’s method [22], the root with the root tip at the forefront is defined as first-order root, its mother root as second-order root, the mother root of the second-order root as third-order root, and so on, until the fourth-order roots are distinguished. Both first-order roots and second-order roots as low-order roots, while third-order roots and fourth-order roots are defined as high-order roots (Fig. 1B). For the retrieved roots, first, measure the fresh weight, analyze their morphological characteristics using a root scanner, calculate the water content and biomass of roots, retain part of the samples for determining the chemical composition of roots at different orders, and finally use the remaining roots for the decomposition experiment. If the amount of roots for decomposition is insufficient, resample by using the above method. Finally, collect the rhizosphere soil within the quadrat for determining the physical and chemical properties of the soil and soil enzyme activities.

The layout stage of the root decomposition experiment (November 2022-September 2023): The root decomposition experiment was set up in November 2022 using the mesh litter bag decomposition method. All collected root samples were naturally air-dried. Subsequently, we determined the mass ratio of different root orders based on field collection conditions and placed them back according to this ratio. Each decomposition bag (15 cm in length and width, 1 mm pore size) was mixed with 3 g of roots, which were divided into two root orders: low-order roots and high-order roots. Points were randomly selected in the four sample plots, buried 10 cm deep, and covered with original soil.

The experiments were as follows(Fig. 1A): (1) Root chemistry change experiment: Root excavated from acid rain, nitrogen deposition and acid rain+nitrogen plots were all placed into the control plot.(The root system framed by the orange marker in the schematic diagram below is processed and then placed into the control plot as indicated by the arrow) (2)Soil environment change experiment: root excavated from the control plot were respectively placed into acid rain, nitrogen deposition and acid rain+nitrogen plots.(The root from control plot marked in blue in the schematic diagram below are placed into the three treatment plots as indicated by the arrows) (3)In-situ experiment: root samples excavated from the control, acid rain, nitrogen deposition and acid rain+nitrogen plots were respectively returned to their original plots.(The in-situ experiments among the four plots are indicated by the green arrows).

In the three sub-experiments, root chemistry change experiment included three types of plots, two root orders, three replicates and five sampling periods, with a total of 90 litterbags; the soil environment change experiment was designed identically to the root chemistry change experiment, also with 90 litterbags; in-situ experiment comprised four types of plots, two root orders, three replicates and five sampling periods, with a total of 120 litterbags. The three sub-experiments amounted to 300 litterbags in total.

During the subsequent 10-month decomposition process, sampling was conducted once every 2 months, with 3 replicates taken from each group to determine indicators such as root mass and pH. In addition, we obtained the monthly average soil moisture and soil temperature data for the months corresponding to the five sampling time points: January: 33.4%, 6.29 °C; March: 35.45%, 5.86 °C; May: 23.51%, 16.64 °C; July: 31.41%, 23.92 °C; September: 30.78%, 22.30 °C. Meanwhile, soil samples in the decomposition bags were collected. We finally obtained samples subjected to 10 different treatments, and the specific types are shown in Table 1.

**Table 1:** The root type under ten treatments obtained from the three experiments

Treatment	Type	Root chemistry	Soil environment
1	In-situ (CK)	CK	CK
2	Root chemistry change	N	CK
3	Root chemistry change	AS	CK
4	Root chemistry change	ASN	CK
5	Soil environment change	CK	N
6	Soil environment change	CK	AS
7	Soil environment change	CK	ASN
8	In-situ	N	N
9	In-situ	AS	AS
10	In-situ	ASN	ASN

### Characterization methods

Root collected in each decomposition stage should first be washed with distilled water to remove the soil and impurities adhering to the roots. Secondly, they are packed into kraft paper bags and placed in an oven to be dried at 60 °C until constant weight is achieved, and the lost mass is determined. The Olson negative exponential decay model is used to describe root decomposition, and the annual decomposition constant K value is calculated.

Total carbon (TC), total nitrogen (TN), and total sulfur (TS) in the soil were determined using an elemental analyzer (Elementar, Germany); the contents of total potassium, calcium, magnesium, iron, manganese, copper, and zinc were measured by atomic absorption spectrometry; The main enzymes involved in the C, N, P, and S cycles were determined spectrophotometrically, following the methods described in previous literature [9, 34, 38], including: sucrase was determined by the 3,5-dinitrosalicylic acid colorimetry method, urease was determined by the sodium benzoate-sodium hypochlorite colorimetry method, acid phosphatase was determined by the disodium p-nitrophenyl phosphate colorimetry method, catalase was determined by the potassium permanganate titration method, aryl sulfatase was determined by the p-nitrophenyl sulfate method, β-glucosidase was determined by the nitrophenol colorimetry method, and N-acetyl-β-D-glucosaminidase enzyme was determined by the p-nitrophenol method. The pH of the soil was determined by the potentiometric method; the available phosphorus (AP) in the soil was determined by the ammonium fluoride-hydrochloric acid extraction method; the available potassium (AK) in the soil was determined by the ammonium acetate-flame photometry method. Nitrate nitrogen was determined by the 2 mol·L⁻¹ KCl extraction-dual-wavelength ultraviolet spectrophotometry method; ammonium nitrogen was determined by the 2 mol·L⁻¹ KCl extraction-indophenol blue colorimetry method. The contents of lignin and cellulose were determined using the improved neutral detergent fiber and acid detergent fiber methods [39].

### Data analysis

We employed one-way ANOVA (SPSS Inc., Chicago, Ill., USA) to examine the differences in root decomposition rates across different root orders among the root chemistry change, soil environment change, and in-situ experiments. R was used to explain the correlation between root decomposition rate, changes in soil nutrients, and changes in root properties. This included a generalized linear model analysis of the root decomposition rate with soil nutrients and root properties, respectively. We employed a Mixed Effects Random Forest (MERF) model implemented in the Python MERF package to conduct SHAP (Shapley Additive Explanations) analysis on the importance of different root and soil indicators to the root decomposition rate. SHAP performs better in complex models with strong nonlinearity and interactive effects [40]. Prior to model training, multicollinearity among input variables was examined using pairwise correlation analysis. In addition, the model employed in this study is relatively robust to multicollinearity, and SHAP values quantify the marginal contribution of each feature to model predictions. A 5-fold cross-validation procedure was employed to evaluate model robustness. The dataset was randomly divided into 5 folds, with first folds used for training and the remaining fold for validation. Model performance metrics were averaged across folds. Hyperparameters of the Random Forest model were optimized using a grid search combined with k-fold cross-validation (k = 5). We systematically evaluated key parameters, including the number of trees (n_ estimators), maximum tree depth (max_ depth), minimum samples required to split a node (min_ samples_ split), minimum samples required at a leaf node (min_ samples_ leaf), and the number of features considered at each split (max_ features). The optimal parameter combination was selected based on the highest cross-validated R². This tuning procedure ensured a balance between model performance and overfitting, improving the robustness and generalizability of the model. Additionally, Origin 2025 (OriginLab Corporation, USA) was used to generate graphs for comparing differences among the root quality change, soil environment change, and “in-situ experiment” groups. The structural equation model (SEM) was used to explain the direct and indirect effects of soil nutrients on enzyme activity, and graphs were plotted using GraphPad Prism 9.0. Microsoft Excel 2021 was used to calculate the cumulative root loss rate.

The cumulative root loss rate (*R*) can be calculated using the following formula:

$$\:R=\left[\right({W}_{0}-{W}_{t})/\:{W}_{0}\:]*100\%$$

Where *W₀* is the initial dry mass of the roots (g), and *Wₜ* is the dry mass of the roots at decomposition time t (g);

The Olson negative exponential decay model was used to describe root decomposition and calculate the annual decomposition constant (*K*):

$$\:{X}_{t}\:/{X}_{0}\hspace{0.17em}=\hspace{0.17em}{e}^{-\hspace{0.17em}Kt}$$

Where *X₀* is the initial dry weight of the roots (g), *Xₜ* is the dry weight of the roots after t time of decomposition (g), *t* is the decomposition time (a, representing “annum”), *K* is the annual decomposition constant.

## Result

### Differences in root decomposition rates under root chemistry change, soil environment change and in-situ experiments

**Fig. 2:**
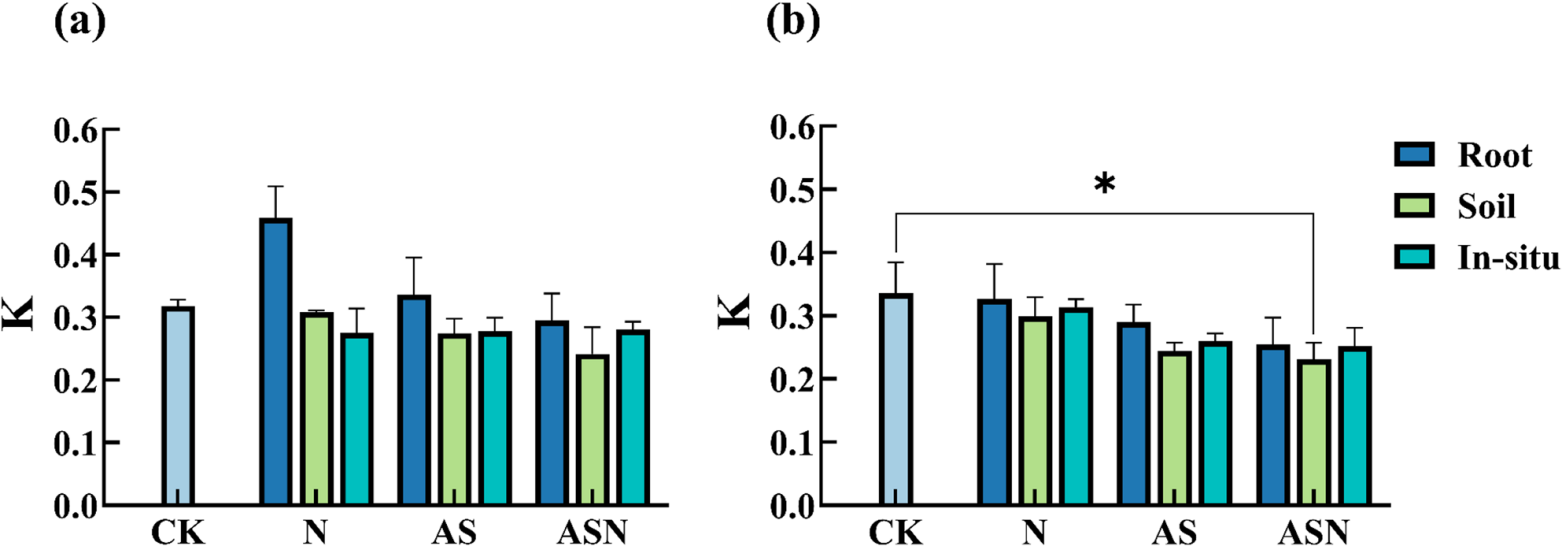
Annual decomposition constant (K) values from three experiments: (1) Root, initial root chemistry, (2) Soil, initial soil environment, and (3) In-situ under acid rain, nitrogen deposition, and their combined effect. Treatments: AS = acid rain; N = nitrogen deposition; ASN = acid rain + nitrogen deposition. **a** low-order roots; (**b**) high-order roots. Asterisks indicate significant differences (*P* < 0.05, *n* = 3)

An analysis of the four treatments (AS, N, ASN, and CK) indicated that nitrogen deposition produced the highest decomposition rate overall: the average decomposition constant K for low-order roots was 0.348 (Fig. 2a), exceeding CK, while high-order roots had an average K of 0.313 (Fig. 2b), slightly lower than CK. By contrast, the ASN treatment had the strongest suppressive effect on decomposition, with K values reduced to 0.274 for low-order roots (Fig. 2a) and 0.246 for high-order roots (Fig. 2b). Root chemistry change, soil environment change, and in-situ experiment analysis showed that root chemistry change exerted the weakest inhibitory effect on decomposition, whereas soil environment changes produced the strongest suppression. Notably, under AS treatment, the decomposition constant K for low-order roots influenced by soil environment change was just 0.241 (Fig. 2a, b). High-order roots decomposed more slowly than low-order roots and exhibited much smaller variations in K across treatments (Fig. 2a, b).

### Effects of different acid and nitrogen treatments on soil environment and root chemistry

**Fig. 3:**
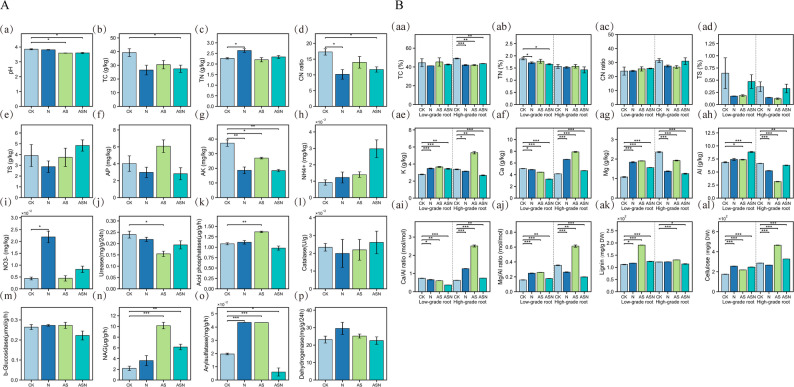
Changes in root chemistry and soil environment under different treatments. **A** soil environment including a, pH; b, soil total carbon (STC); c, soil total nitrogen (STN); d, soil carbon: nitrogen ratio (SC: N); e, soil total sulfur (STS); f, available phosphorus (AP); g, Available Potassium (AK); h, ammonium nitrogen (NH_4_^+^); i, nitrate nitrogen (NO_3_^−^); j, Urease; k, Acid phosphatase; l, Catalase; m, β-Glucosidase (BG); n, N-acetyl-b-glucosaminidase (NAG); o, Arylsulfatase; p, Dehydrogenase. **B** root chemistry, including aa, root total carbon (RTC); ab, root total nitrogen (RTN); ac, root carbon: nitrogen ratio (RC: N); ad, root total sulfur (RTS); ae, Potassium (K); af, Calcium (Ca); ag, Magnesium (Mg); ah, Aluminum (Al); a.i., Calcium: Aluminum Ratio (Ca: Al); aj, Magnesium: Aluminum Ratio (Mg: Al); ak, Lignin; al, Cellulose. CK, control; N, nitrogen deposition treatment; AS, acid rain treatment; ASN, nitrogen deposition +acid rain treatment; _*_ represents *P* < 0.05, _**_ represents *P* < 0.01, and _***_ represents *P* < 0.001

Both acid rain and nitrogen deposition significantly altered soil conditions. Acid rain lowered soil pH and modified enzyme activities: Urease activity decreased to 64.4% of CK (*P* < 0.05; Fig. 3j), whereas acid phosphatase and arylsulfatase activities increased by 26.9% and 120.3%, respectively (*P* < 0.05; Fig. 3k, o). Nitrogen deposition affected nutrient availability: NO_3_^−^ increased by 403.2% after addition (*P* < 0.05; Fig. 3i), while AK declined to 0.019 g kg^− 1^ (Fig. 3g).

Similarly, root chemistry was substantially affected by acid and nitrogen deposition, with the exception of RTS, RTN, and RTC (Fig. 3B). Acid rain increased lignin and cellulose contents: lignin in low-order roots rose to 191 g kg^− 1^ DW, an increase of 71.67% (*P* < 0.05; Fig. 3ak), and cellulose in high-order roots increased to 464.79 g kg^− 1^ DW, an increase of 62.56% (*P* < 0.05; Fig. S3al). Nutrient concentrations in low-order roots increased under both treatments, with acid rain producing the largest effects: Potassium (K), Magnesium (Mg), and Aluminum (Al) increased by 0.87 g kg^− 1^, 0.82 g kg^− 1^, and 0.49 g kg^− 1^, respectively (*P* < 0.05; Fig. 3ae, ag, ah). In contrast, high-order roots under nitrogen deposition showed significant decreases in K, Mg, and Al by 0.22 g kg^− 1^, 0.99 g kg^− 1^, and 1.40 g kg^− 1^, respectively.

### Responses of decomposition constant K of different root orders to root chemistry

**Fig. 4:**
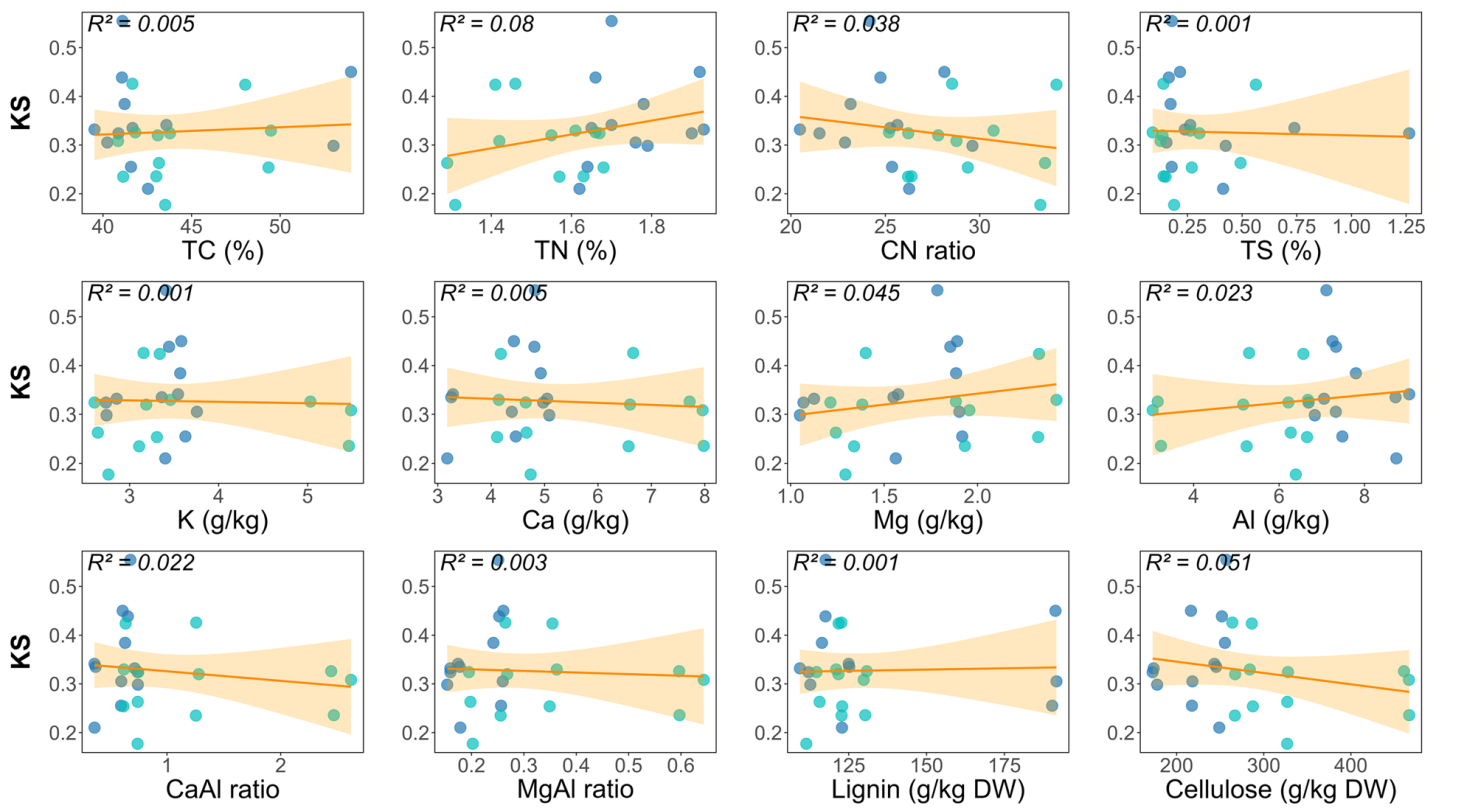
Responses of the decomposition constant (K) of total roots to root chemistry under acid rain and nitrogen deposition. Elements in the figure include: root total carbon (RTC); root total nitrogen (RTN); root carbon: nitrogen ratio (RC: N); root total sulfur (RTS); potassium (K); calcium (Ca); magnesium (Mg); aluminum (Al); Ca: Al ratio (Ca: Al); Mg: Al ratio (Mg: Al); lignin; cellulose. Independent sample size: *N* = 24

There is a correlation between root chemistry and root decomposition constants. Acid rain and N deposition increased K, Mg and Al in low-order roots (*P* < 0.05) (Fig. S4). Among them, the root decomposition constant of low-order roots showed an increasing trend with increase in Ca: Al (*R*^*2*^ = 0.112, *P* = 0.289) and Mg: Al ratios (*R*^*2*^ = 0.219, *P* = 0.125) (Fig. S4). In contrast, the AS and N treatment reduced Al and increased Ca in high-order roots (Al decreased by 3.49 g kg^− 1^ and 1.40 g kg^− 1^, respectively, while Ca increased by 1.88 g kg^− 1^ and 0.63 g kg^− 1^, respectively.) (Fig. S5), but neither Ca nor Al showed clear correlation with root decomposition of high-order roots. (Fig. S5).Roots of different orders had similar RTN and Mg concentrations (Fig. 4). Differences in Ca: Al ratio and Mg: Al ratios were also apparent (Fig. S4, S5). Together, these chemical indicators can help distinguish the functional ecology of different root orders.

### Responses of decomposition constant K of different root orders to soil environment

Generalized linear models (GLMs) were used to analyze the relationships between the decomposition constant (K) of both low-order and high-order roots and various soil properties. Soil pH was significantly positively correlated with root decomposition constants (*R*^*2*^ = 0.341, *P* < 0.01). Meanwhile, root decomposition constants were closely related to carbon and nitrogen cycling processes. For example, NH_4_^+^ (*R*^*2*^= 0.281, *P* < 0.01) and NAG (*R*^*2*^= 0.184, *P* < 0.05) were both significantly negatively correlated with root decomposition constants. In contrast, AK in soil nutrients was significantly positively correlated with root decomposition constants (*R*^*2*^= 0.234, *P* < 0.05). Moreover, root decomposition constants tended to increase with increasing urease activity (*R*^*2*^= 0.127, *P* = 0.08) and soil total carbon (STC) (*R*^*2*^= 0.132, *P* = 0.08).

### Differences in the contributions of root chemistry and soil environment to the decomposition of roots with different root orders and their influencing pathways

In the simulated in-situ experiment, the decomposition rate of low-order roots (represented by the mean value of treatments involving changes in root chemistry and soil environment) was consistently higher than that in the in-situ treatment (The increases in the five periods were 0.001, 0.020, 0.037, 0.035, and 0.020, respectively) (*P* > 0.05). By contrast, decomposition rates in all five simulated in-situ experiments for high-order roots were lower than those recorded in in-situ experiment (The decreases in the five periods were 0.019, 0.009, 0.005, 0.030, and 0.021, respectively) (*P* > 0.05). Under the synergistic effects of root chemistry and soil environment in the in-situ experiment, the decomposition rate of low-order roots was consistently lower than that in the simulated in-situ experiment, showing a trend of initial enhancement followed by weakening of inhibition (Fig. 6B). In contrast, the decomposition rate of high-order roots was higher in the in-situ experiment than in the simulated in-situ experiment, suggesting that root chemistry and soil environment tend to mutually alleviate their inhibitory effects. (Fig. 6B). Although the interaction between root chemistry and soil environment differed between root orders, both of them transitioned from initially stronger suppression by root chemistry change in the short term to stronger suppression driven by soil environment change over the long term. For low-order roots, this shift in the dominant factor occurred in the sixth month (The mean decomposition rate was 0.347 for the root chemistry experiment and 0.324 for the soil-environment experiment), while for high-order roots, it occurred in the tenth month (The mean decomposition rate was 0.385 for the root chemistry experiment and 0.371 for the soil-environment experiment). Over time, suppression gradually weakened and eventually returned to near the original decomposition rate (Fig. 6A).

Random Forest Model performance was evaluated using the coefficient of determination (R²) and root mean square error (RMSE). The model achieved an R² of 0.59 and an RMSE of 0.92, indicating good predictive accuracy, which was robust to conduct the SHAP analysis to reveal the feature importance. The structural equation model was constructed based on Table 2. The results of path analysis and SHAP analysis indicate that nutrient structure played the most critical role in both cases: for high-order roots, soil C: N (SC: N) was the dominant factor, with a direct effect of 0.38; whereas for low-order roots, available potassium (AK) was the leading factor, with a direct effect of 0.57 (Fig. 6C, D). Even after ten months, microbial activity and cellulose continued to influence root decomposition.

**Table 2:** Standard values of structural equation model fitting index of roots

Abbreviation	Low-order	High-order	Acceptance criteria
*χ²*	0.072	0.114	
DF	1	1	
*P*-value	0.789	0.735	> 0.05
GFI	0.99	0.99	> 0.90
RMSEA	0.01	0.01	< 0.08

## Discussion

### Effects of soil environmental changes and root chemical variations on root decomposition

Root decomposition is closely regulated by soil environment and root chemistry [41, 43]. Acid rain and nitrogen deposition, as key drivers of soil environmental change, can alter soil nutrient structure, affects plant growth and metabolic processes, thereby inducing root chemistry changes and further regulating root decomposition [44]. In this study, pH significantly influence root decomposition rates by modifying root chemistry—reducing root calcium (Ca) content and increasing cellulose content (Fig. 3a, 3af, al)—which is consistent with Wang’s findings [45] on the effects of soil acidification on root decomposition in alpine grasslands. Moreover, acid rain exerts a significant effect on soil enzyme activities (Fig. 3A), thereby influencing the composition of soil microbial communities. The impacts of different community compositions on root decomposition differ significantly. When dominant decomposers are present in the soil, root decomposition is significantly accelerated; conversely, decomposition may be limited or even inhibited [46]. Microbial communities undergo succession during different stages of root decomposition as well: bacteria dominate in the early stage, while fungi such as saprotrophic fungi become dominant in the later stage [47]. Different microbial communities produce distinct types of decomposing enzymes, which directly determine the type and rate of organic matter degradation [47]. Differences in their utilization efficiency for various chemical components in roots directly influence the pattern and rate of root decomposition [46]. What’s more, owing to the inherent differences among various root systems and their associated microorganisms, their adaptability to the environment cannot be fully consistent. Therefore, the experimental design still has certain limitations regarding its independence.

**Fig. 5:**
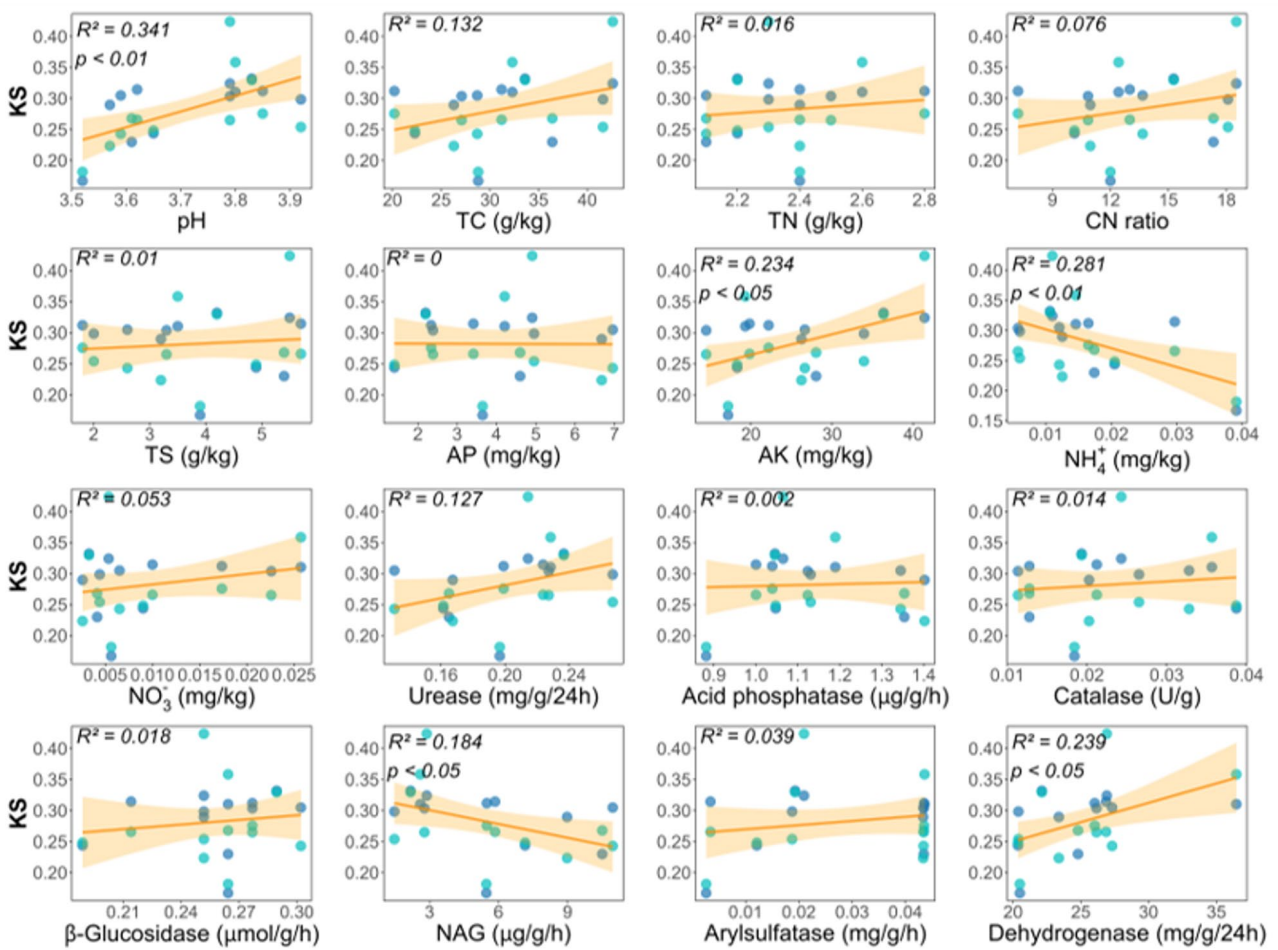
Responses of the decomposition constant (K) of total roots to soil environmental factors under simulated acid rain and nitrogen deposition. Each panel shows the relationship between K and a specific soil variable: pH; soil total carbon (STC); soil total nitrogen (STN); soil carbon: nitrogen ratio (C: N); soil total sulfur (STS); available phosphorus (AP); available potassium (AK); ammonium nitrogen (NH_4_^+^); nitrate nitrogen (NO_3_^−^); urease activity; acid phosphatase activity; catalase activity; β‑glucosidase (BG) activity; N‑acetyl‑β‑glucosaminidase (NAG) activity; arylsulfatase activity; dehydrogenase activity. Independent sample size: *N* = 24

**Fig. 6:**
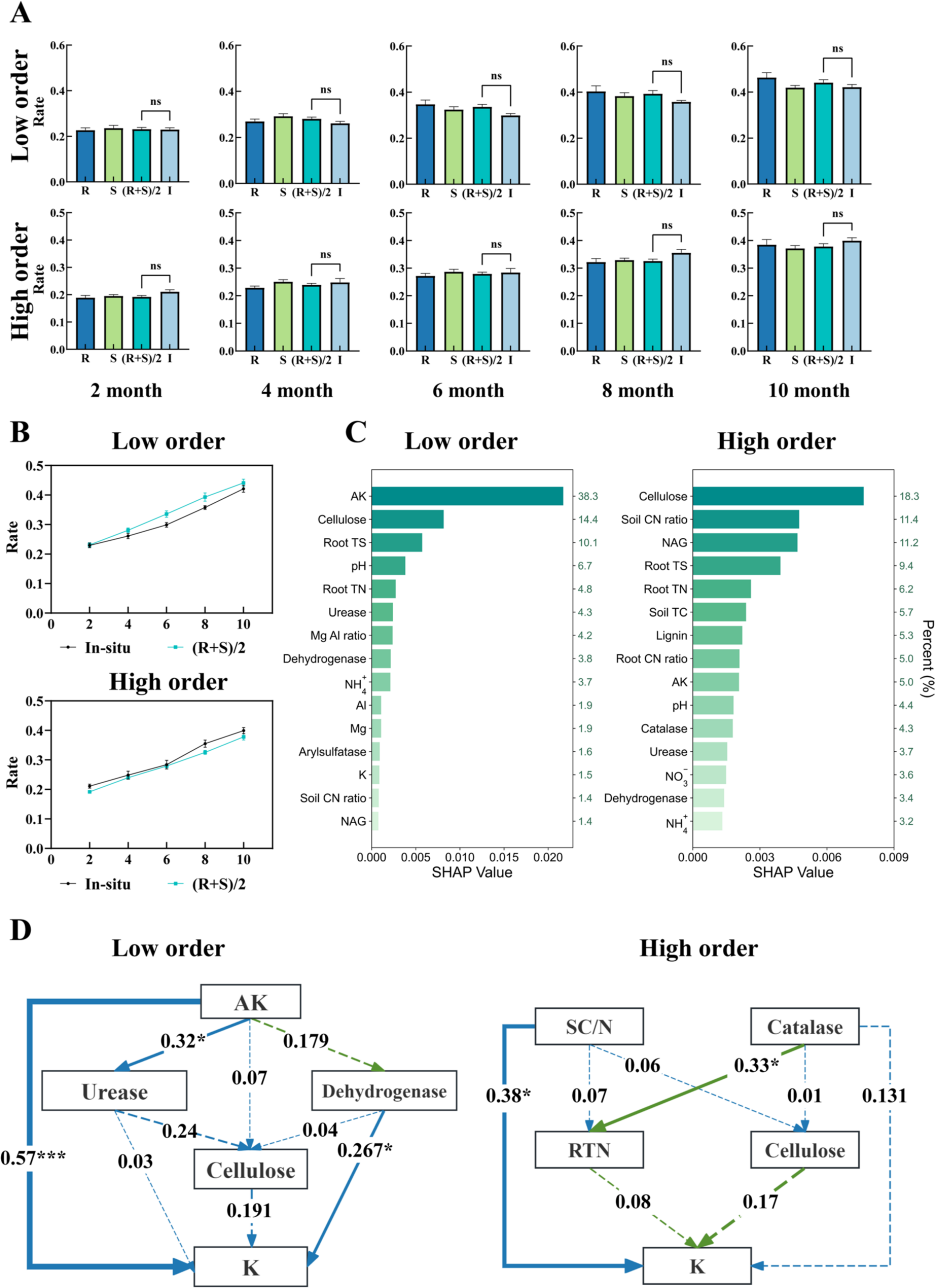
Contribution of Root chemistry Changes and Soil Environment Changes to Root Decomposition, and the Key Driving Factors. (**A**) The decomposition rates of two root orders in three experiments. R, root chemistry change experiment; S, soil environment change experiment; (R + S)/2, the average value of the annual root decomposition constant of root chemistry changes and soil environment changes; I, In-situ experiment. (**B**) Trend of Root decomposition rates in five stages for two root orders. (**C**) Importance of different root and soil indicators to the root decomposition rate. (**D**) Paths of key soil and root indicators on root decomposition

Soil nitrogen (N) dynamics also plays an important role in our study. N treatment significantly increases soil total nitrogen content while reducing the soil carbon-to-nitrogen ratio (SC: N) (Fig. 3b, c, d). This change in soil N availability can alter the root carbon-to-nitrogen ratio (RC: N) and influence carbon accumulation in belowground biomass [39], thereby exerting either promotional [48] or inhibitory [49, 51] effects on root decomposition. Notably, SHAP analysis and GLMs further confirmed that SC: N is the most critical factor influencing the long-term decomposition of high-order roots, and exogenous nitrogen addition significantly lowers SC: N (Fig. 6C). Interestingly, nitrogen primarily regulates root decomposition in the form of ammonium nitrogen (NH₄⁺) rather than total nitrogen, as excess nitrogen generated by microorganisms during decomposition mainly exists as NH₄⁺ (Fig. 5). In this study, the model showed moderate explanatory power (R^2^= 0.59), reflecting the complexity of fine root decomposition. Decomposition is affected by various factors including soil microbial community composition, soil temperature and moisture, mycorrhizal type. These uncertainties limit predictive performance. This study aims to identify key drivers and quantify their relative importance rather than maximize accuracy. Consistent feature contributions from the model support the reliability of the results.

GLMs further validates the direction and significance of key relationships detected by machine learning. Consistent results from both methods improve confidence and strengthen mechanistic understanding of fine root decomposition.

### Effects of different root order differences on root decomposition processes

Root order differences significantly modulate root decomposition processes by shaping root chemical properties, lifespan, and structural characteristics. Low-order roots, characterized by higher nitrogen content, lower RC: N (Fig. 3ac), shorter lifespan, and simpler structure, show greater decomposition rate dispersion in GLMs (Fig. S4). Contrary to the view proposed by Jing [52] that “decomposers can only utilize litter with a low RC: N,” low-order roots in our study were not necessarily more easily decomposed, which is associated with their higher acid-insoluble compound content [6].

In contrast, high-order roots—located farther from the root tip—have larger diameters, lower N content, longer lifespans [53], and more stable decomposition characteristics: their decomposition rate variation was more consistent with that of total roots (Fig. S5). Furthermore, the high nitrogen content and low lignin content of lower-order roots directly result in their low lignin-to-nitrogen (Lignin: N) ratio. Numerous studies have demonstrated that root decomposition rate is negatively correlated with Lignin: N ratio, which is one of the crucial factors contributing to the rapid decomposition of lower-order roots [31, 54]. SHAP analysis shows no significant difference in the contribution of root chemistry between low-order roots and high-order roots (Fig. 6C, D). Numerous studies have demonstrated that root tissue density increases with rising root order, and denser root tissues are hard to decompose [55]. This is further confirmed by the stronger internal correlations among lignin, cellulose, Al and Mg in high-order roots in the study (Fig. S2a, b), as the tight associations of these chemical components regulate the resistance of high-order roots to decomposition.

Besides differences in root order, several other factors can significantly affect root decomposition.

Mycorrhizal fungi can also slow root decomposition by altering root chemical composition, such as increasing phenolic compounds [56] or suppress decomposer activity via nutrient competition such as nitrogen [57]. Even after death, their residual mycelia continue to influence decomposition dynamics [46]. Fine roots of different mycorrhizal types, such as arbuscular mycorrhizal (AM) and ectomycorrhizal (ECM) roots exhibit distinct decomposition rates [58], with ECM roots decompose more slowly than AM roots generally [59]. Mycorrhizal colonization is higher in low-order roots and lower in high-order roots, which may also potentially influence root decomposition rates [24, 60]. Therefore, the absence of observations on mycorrhizal colonization constitutes one of the limitations of this study. Soil moisture and temperature interactions strongly influence root decomposition: at 60%-80% field capacity, elevated temperature significantly accelerates decomposition [61], while extreme moisture conditions restrict microbial metabolism and reduce decomposition rates [62]. Soil water content affects pore aeration, with anaerobic conditions inhibiting decomposition [63]. As key abiotic factors, temperature and moisture regulate enzyme activity, microbial community structure, and aeration, thereby influencing root organic matter transformation [63, 65]. With the intensification of global change, they will jointly affect the root decomposition process along with acid rain and nitrogen deposition. Therefore, future research should consider the impacts of multiple global change factors on root decomposition.

## Conclusion

Over nearly two years, this study examined how soil environment and root chemistry jointly influence decomposition rate of different root orders under the context of conditions simulating acid rain and nitrogen deposition. Three experiments, namely root chemistry change, soil environment change, and in-situ experiment had been carried out. The results showed that soil environment had the strongest inhibitory effect on root decomposition. SHAP analysis indicated that soil environment predictive importance accounted for 61.2% for low-order roots and 55.9% for high-order roots. The decomposition process was dominated by the root chemistry in the short term and then shifted to being dominated by long-term soil environmental factors, among which soil nutrients such as available potassium (The relative contribution accounted for 38.3%) and soil carbon- nitrogen ratio (The relative contribution accounted for 11.4%) were the important driving factors. During this process, the duration of the inhibitory effect dominated by the chemistry of high-order roots was longer than that of low-order roots (6 months for low-order roots and 10 months for high-order roots). In addition, across the five sampling periods, root chemistry and soil environment showed a trend of synergistic inhibition in the decomposition of lower-order roots, whereas they exhibited a trend of mutually weakening inhibitory effects in higher-order roots. Notably, the changing trends of calcium: aluminum ratio (Ca: Al) and magnesium-to-aluminum ratio (Ma: Al) of the two types of roots were opposite, which may serve as one of the important indicators for distinguishing roots of different orders.

Overall, this study emphasizes that the impact on the root decomposition process is not a simple superposition of soil environment and root chemistry. In the in-situ experiment their effects may either be enhanced or weakened—and this result is likely dependent on the response strategies of roots of different orders, while changes in the soil environment will have a more long-term impact on root decomposition. These findings help us further understand the decomposition process of roots of different orders in forest ecosystems.

## Supplementary Information

Supplementary Material 1.

## Data Availability

Data is provided within the manuscript or supplementary information files.
