# Supplementary material for: Soil environmental changes drive root decomposition under acid and nitrogen deposition in Chinese fir plantation, China
Source: BMC Plant Biol. 2026 Apr 25;26:995. doi: 10.1186/s12870-026-08816-z (PMC13251174; doi:10.1186/s12870-026-08816-z)
Supplement: Supplementary file 1 — Supplementary Material 1. [file 12870_2026_8816_MOESM1_ESM.docx]

**Soil environmental changes drive root decomposition under acid and nitrogen deposition in Chinese fir plantation, China**

Xuanran Yu ^a^, Chang Pan ^b^, Jinchi Zhang ^a, *^, Xingyu Zhang ^a^, Aodeng Rong ^c^, Qingguo Tong ^d^, Xiongfei Zhang ^a^, Chong Li ^e^, Hui Nie ^a^, Jingyi Zeng ^a^, Yangyang Wang ^a^, Xin Liu ^a, *^

^a^ Co-Innovation Center for Sustainable Forestry in Southern China of Jiangsu Province, Key Laboratory of Soil and Water Conservation and Ecological Restoration of Jiangsu Province, Nanjing Forestry University, Nanjing 210037, China

^b^ State Key Laboratory of Nutrient Use and Management, College of Resources and Environmental Sciences, China Agricultural University, Beijing 100193, China

^c^ Inner Mongolia Big Data Center, Hohhot 010090, China

^d^ CNBM Mining Investment (Jiangsu) Co., Ltd.

^e^ Department of Renewable Resources, University of Alberta, Edmonton, AB T6G 2E3, Canada

*Corresponding Author: Jinchi Zhang ([zhang8811@njfu.edu.cn](mailto:zhang8811@njfu.edu.cn))

*Corresponding Author: Xin Liu ([liuxinswc@njfu.edu.cn](mailto:liuxinswc@njfu.edu.cn))

**Email and ORCID:**

Xuanran Yu: yuxuanran@njfu.edu.cn (0009-0004-4296-3355);

Chang Pan: [kevinpc2002@163.com](mailto:kevinpc2002@163.com);

Jinchi Zhang *: [zhang8811@njfu.edu.cn](mailto:zhang8811@njfu.edu.cn) (0000-0002-0517-7214);

Xingyu Zhang: [zhangxingyu@njfu.edu.cn](mailto:zhangxingyu@njfu.edu.cn);

Aodeng Rong: [147016617@qq.com](mailto:147016617@qq.com);

Qingguo Tong: 451281402@qq.com;

Xiongfei Zhang: zxf15180113449@njfu.edu.cn (0009-0004-9285-9038);

Chong Li: cli5104@njfu.edu.cn (0000-0001-9330-5396);

Hui Nie: huinie948@gmail.com (0000-0003-2980-8815);

Jingyi Zeng: [zjy0135@njfu.edu.cn](mailto:zjy0135@njfu.edu.cn);

Yangyang Wang: 1162647510@njfu.edu.cn;

Xin Liu *: liuxinswc@njfu.edu.cn (0000-0001-8641-7170)


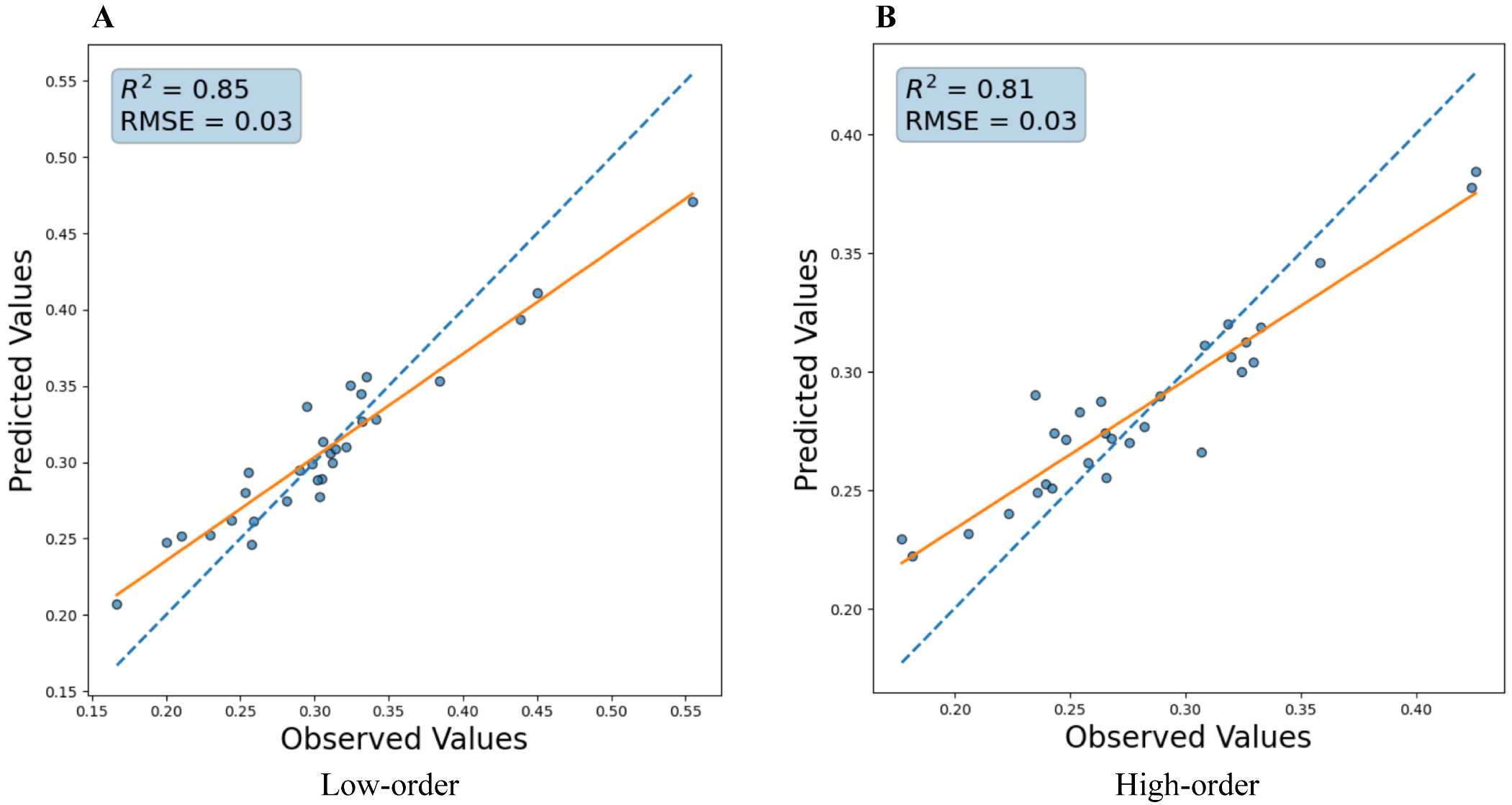


Figure.S1 SHAP performance evaluation. A, Low-order; B, High-order.


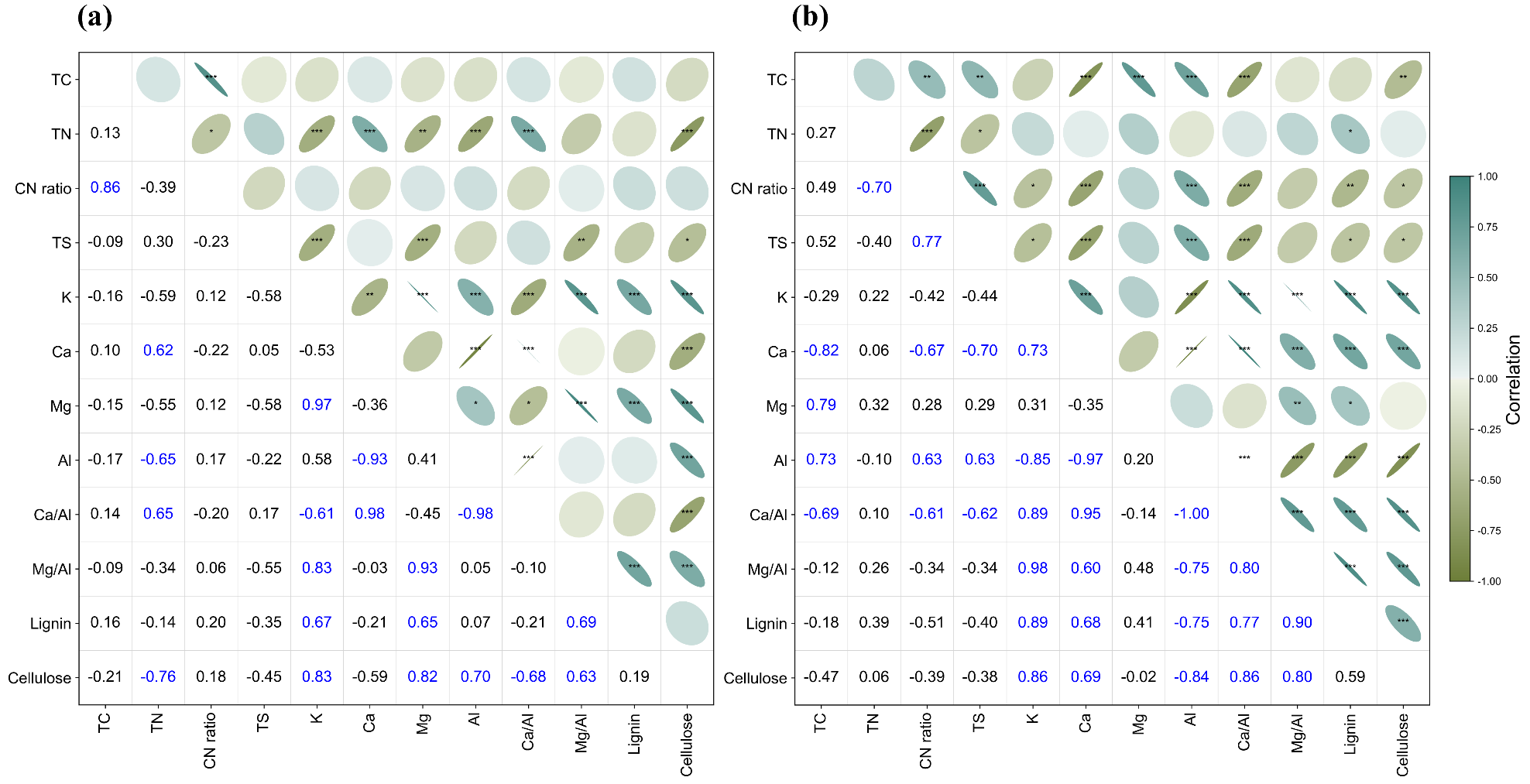


**Fig. S2 Correlation between root chemistry factors of high-order roots and low-order roots mediated by acid rain and nitrogen deposition.** a, correlation of low-order roots; b, correlation of high-order roots. The pairwise correlations of these variables are displayed with a color gradient, indicating Pearson's correlation coefficients. The contents of plant elements include Root total carbon (RTC), Root total nitrogen (RTN), Root carbon: nitrogen ratio(RC: N), Root total sulfur (RTS), Potassium (K), Calcium (Ca), Magnesium (Mg), Aluminum (Al), Calcium: aluminum ratio (Ca: Al), Magnesium: aluminum ratio (Mg: Al), Lignin, Cellulose. * indicates a significant correlation between pairwise elements (*P*<0.05). * represents *P*<0.05, ** represents *P*<0.01, and *** represents *P*<0.001.


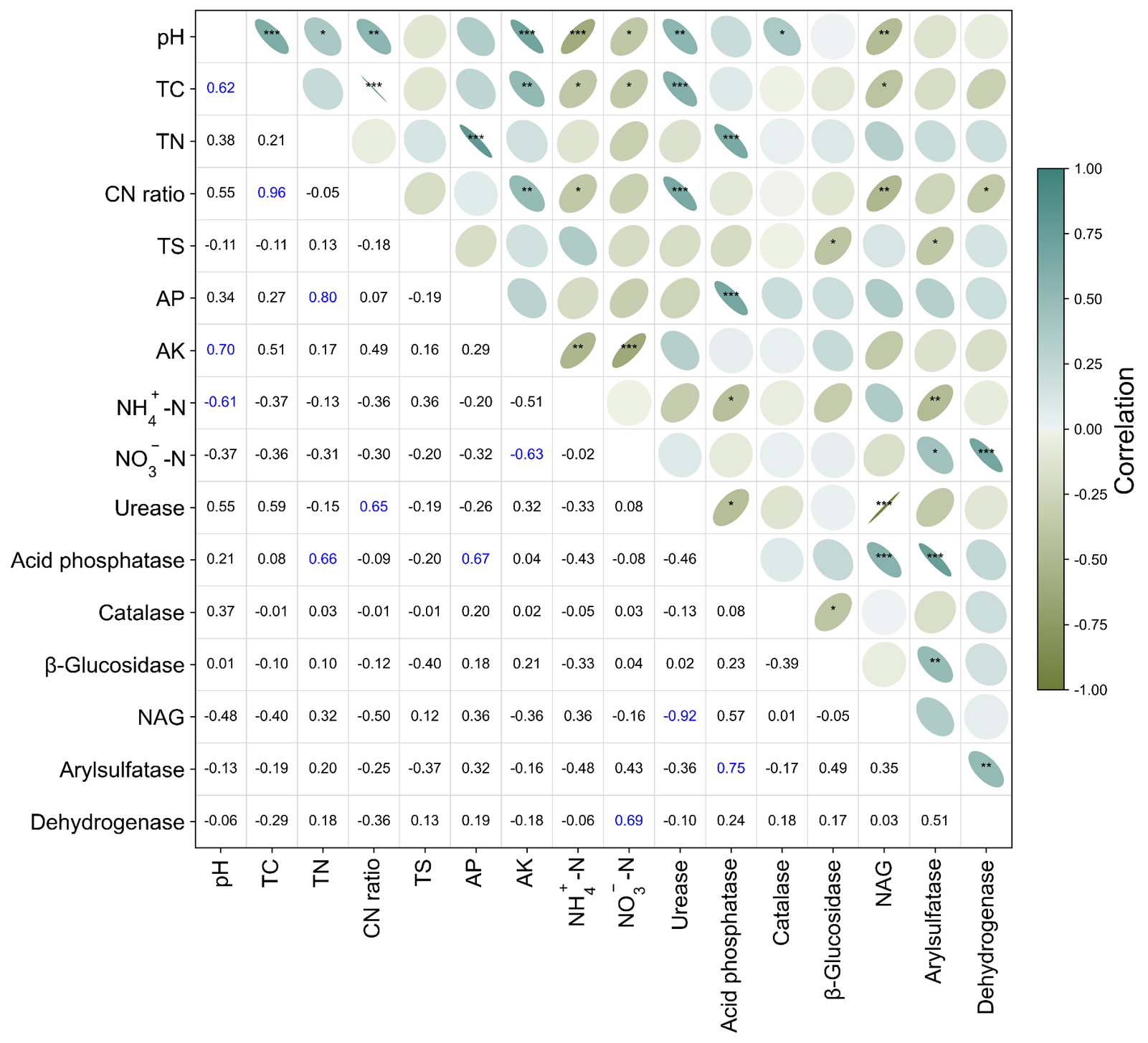


**Fig. S3 Correlation between soil environmental factors mediated by acid rain and nitrogen deposition.** The pairwise correlations of these variables are displayed with a color gradient, indicating Pearson's correlation coefficients. The soil physical and chemical properties include pH, Soil total carbon (STC), Soil total nitrogen (STN), Soil carbon: nitrogen ratio (SC: N), Soil total sulfur (STS), Available potassium (AK), Available phosphorus (AP), Ammonium nitrogen (NH₄⁺-N), Nitrate nitrogen (NO₃⁻-N), Urease, Acid phosphatase (ACP), Catalase, β-Glucosidase, NAG, Arylsulfatase and Dehydrogenase. * indicates significant correlation between pairwise elements (*P*<0.05). * represents *P*<0.05, ** represents *P*<0.01, and *** represents *P*<0.001.





**Fig. S4. Responses of the decomposition constant (K) of low-order roots to root chemistry under acid rain and nitrogen deposition.** Elements in the figure include: Root total carbon (RTC); Root total nitrogen (RTN); Root carbon: nitrogen ratio (RC: N); Root total sulfur (RTS); Potassium (K); Calcium (Ca); Magnesium (Mg); Aluminum (Al); Calcium: Aluminum ratio (Ca: Al); Magnesium: Aluminum ratio (Mg: Al); Lignin; Cellulose. Independent sample size: N = 12.


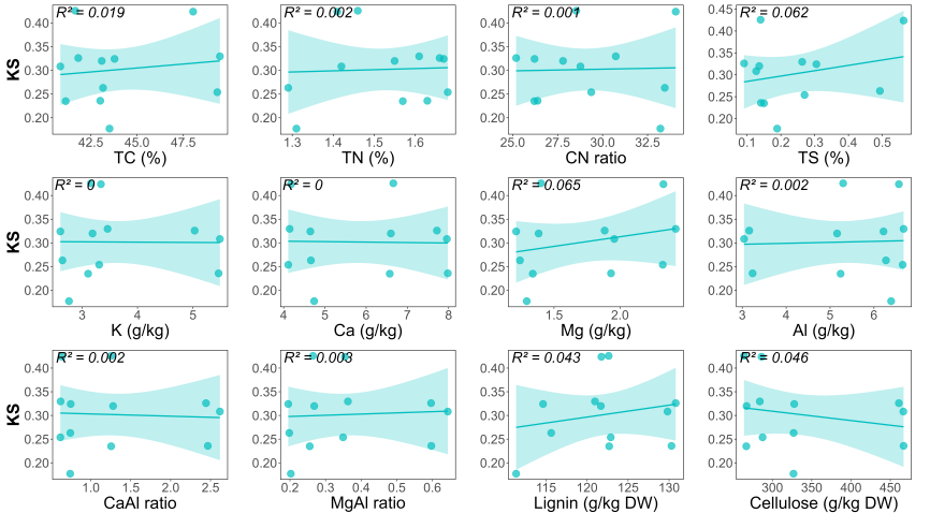


**Fig. S5. Responses of the decomposition constant (K) of high-order roots to root chemistry under acid rain and nitrogen deposition.** Elements in the figure include: Root total carbon (RTC); Root total nitrogen (RTN); Root carbon: nitrogen ratio (RC: N); Root total sulfur (RTS); Potassium (K); Calcium (Ca); Magnesium (Mg); Aluminum (Al); Calcium: Aluminum ratio (Ca: Al); Magnesium: Aluminum ratio (Mg: Al); Lignin; Cellulose. Independent sample size: N = 12.





**Figure S6. Responses of the decomposition constant (K) of low-order roots to soil environmental factors under simulated acid rain and nitrogen deposition.** Elements in the figure include: pH; Soil total carbon (STC); Soil total nitrogen (STN); Soil carbon: nitrogen ratio (C: N); Soil total sulfur (STS); Available phosphorus (AP); Available potassium (AK); Ammonium nitrogen (NH_4_^+^); Nitrate nitrogen (NO_3_^−^); Urease activity; Acid phosphatase activity; Catalase activity; β‑glucosidase (BG) activity; N‑acetyl‑β‑glucosaminidase (NAG) activity; Arylsulfatase activity; Dehydrogenase activity. Independent sample size: N = 12.

**
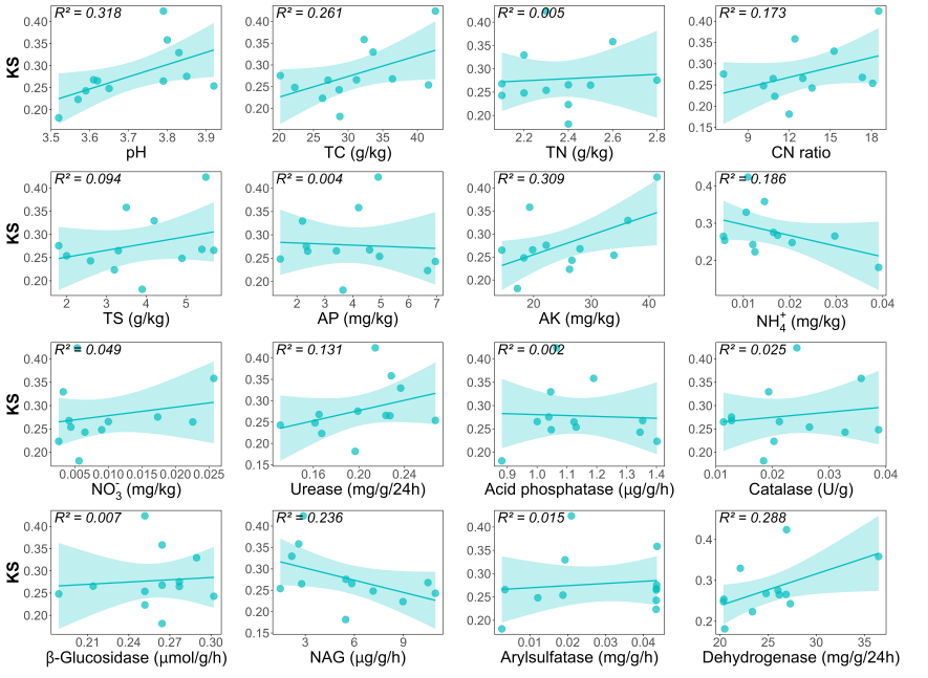
**

**Figure S7. Responses of the decomposition constant (K) of high-order roots to soil environmental factors under simulated acid rain and nitrogen deposition.** Elements in the figure include: pH; Soil total carbon (STC); Soil total nitrogen (STN); Soil carbon: nitrogen ratio (C: N); Soil total sulfur (STS); Available phosphorus (AP); Available potassium (AK); Ammonium nitrogen (NH_4_^+^); Nitrate nitrogen (NO_3_^−^); Urease activity; Acid phosphatase activity; Catalase activity; β‑glucosidase (BG) activity; N‑acetyl‑β‑glucosaminidase (NAG) activity; Arylsulfatase activity; Dehydrogenase activity. Independent sample size: N = 12.

| Low-order | | | | | | | | | | | | | | |
| --- | --- | --- | --- | --- | --- | --- | --- | --- | --- | --- | --- | --- | --- | --- |
|  | pH | STC | STN | SC/N | STS | AP | AK | NH_4_^+^ | NO_3_^-^ | Urease | Acid phosphatase | Catalase | β-Glucosidase | NAG |
| R^2^ | 0.394 | 0.031 | 0.041 | 0.009 | 0.031 | 0.005 | 0.162 | 0.46 | 0.062 | 0.13 | 0.029 | 0.005 | 0.045 | 0.135 |
| P | ＜0.05 | 0.583 | 0.526 | 0.764 | 0.582 | 0.833 | 0.194 | ＜0.05 | 0.434 | 0.25 | 0.594 | 0.828 | 0.51 | 0.24 |
|  | Arylsulfatase | Dehydrogenase | RTC | RTN | RC/N | RTS | K | Ca | Mg | Al | Ca/Al | Mg/Al | Lignin | Cellulose |
| R^2^ | 0.094 | 0.193 | 0.007 | 0.032 | 0.002 | 0.065 | 0.023 | 0.134 | 0.104 | 0.092 | 0.112 | 0.219 | 0.012 | 0.11 |
| P | 0.333 | 0.153 | 0.8 | 0.577 | 0.885 | 0.425 | 0.641 | 0.242 | 0.306 | 0.338 | 0.289 | 0.125 | 0.734 | 0.292 |

| High-order | | | | | | | | | | | | | | |
| --- | --- | --- | --- | --- | --- | --- | --- | --- | --- | --- | --- | --- | --- | --- |
|  | pH | STC | STN | SC/N | STS | AP | AK | NH_4_^+^ | NO_3_^-^ | Urease | Acid phosphatase | Catalase | β-Glucosidase | NAG |
| R^2^ | 0.318 | 0.261 | 0.005 | 0.178 | 0.094 | 0.004 | 0.309 | 0.186 | 0.049 | 0.131 | 0.002 | 0.025 | 0.007 | 0.236 |
| P | 0.056 | 0.0897 | 0.819 | 0.178 | 0.332 | 0.845 | 0.061 | 0.161 | 0.49 | 0.247 | 0.882 | 0.626 | 0.801 | 0.11 |
|  | Arylsulfatase | Dehydrogenase | RTC | RTN | RC/N | RTS | K | Ca | Mg | Al | Ca/Al | Mg/Al | Lignin | Cellulose |
| R^2^ | 0.015 | 0.288 | 0.019 | 0.002 | 0.001 | 0.062 | 0 | 0 | 0.065 | 0.002 | 0.002 | 0.003 | 0.043 | 0.046 |
| P | 0.707 | 0.0718 | 0.665 | 0.89 | 0.927 | 0.437 | 0.977 | 0.949 | 0.425 | 0.903 | 0.878 | 0.863 | 0.516 | 0.503 |

**Table.S1. Relationships and significance levels between decomposition constants (K) of roots of different orders, soil environment, and root chemistry.** Generalized Linear Models (GLMs) were used for curve fitting. soil total carbon (STC); soil total nitrogen (STN); soil carbon-nitrogen ratio (SC/N); soil total sulfur (STS); available phosphorus (AP); Available Potassium (AK); ammonium nitrogen (NH_4_^+^); nitrate nitrogen (NO_3_^-^); N-acetyl-b-glucosaminidase (NAG); root total carbon (RTC); root total nitrogen (RTN); root carbon-nitrogen ratio (RC/N); root total sulfur (RTS); Potassium (K); Calcium (Ca); Magnesium (Mg); Aluminum (Al); Calcium-Aluminum Ratio (Ca/Al); Magnesium-Aluminum Ratio (Mg/Al).


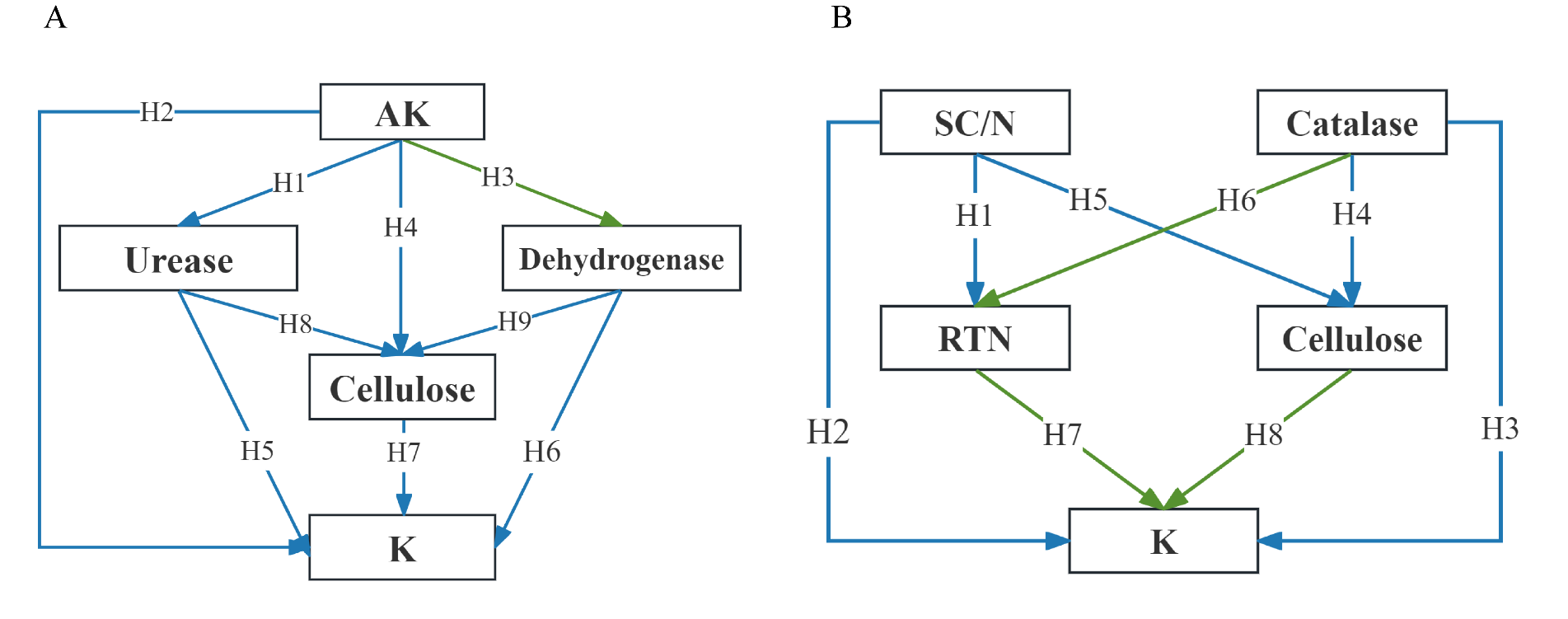


**Figure S8. Conceptual diagram. Low-order:** H1: AK has a positive effect on Urease; H2: AK has a positive effect on K; H3: AK has a negative effect on Dehydrogenase; H4: AK has a positive effect on Cellulose; H5: Urease has a positive effect on K; H6: Dehydrogenase has a positive effect on K; H7: Cellulose has a positive effect on K; H8: Urease has a positive effect on Cellulose; H9: Dehydrogenase has a positive effect on Cellulose. High-order: H1: SC/N has a positive effect on RTN; H2: SC/N has a positive effect on K; H3: Catalase has a positive effect on K; H4: Catalase has a positive effect on Cellulose; H5: SC/N has a positive effect on Cellulose; H6: Catalase has a negative effect on RTN; H7: RTN has a negative effect on K; H8: Cellulose has a negative effect on K. The construction of SEM is based on SHAP analysis and model parameters.
